# Systemic inflammation, lifestyle behaviours and dementia: A 10‐year follow‐up investigation

**DOI:** 10.1002/alz.095417

**Published:** 2025-01-09

**Authors:** Leah Hilary, Philipp Frank, Dorina Cadar

**Affiliations:** ^1^ Behavioural Science and Health, University College London, London United Kingdom; ^2^ Centre for Dementia Studies, Brighton and Sussex Medical School, Brighton United Kingdom; ^3^ University College London, Institute of Epidemiology and Health Care, London United Kingdom

## Abstract

**Background:**

Lifestyle behaviours have been linked to dementia incidence, but their cumulative impact on dementia and the underlying mechanisms remain poorly understood. This study investigated the association of co‐occurring lifestyle behaviours with dementia incidence and the mediating role of systemic inflammation in this association.

**Method:**

The sample comprised 3,131 participants (55.2% female) from the English Longitudinal Study of Ageing aged 52‐92 years at baseline (2008/09). Self‐reported baseline lifestyle behaviours (alcohol intake, fruit and vegetable consumption, smoking, physical activity, sleep duration, social engagement, and cognitive activity) were summed to derive an index of lifestyle behaviours, ranging from 0 to 7, with higher scores denoting a higher number of health‐risk behaviours. Incident dementia cases (n = 130, 4.2%) were identified through doctor‐diagnosed dementia, informant interviews, and health records between 2014/15 and 2018/19. Systemic inflammation was measured through fasting plasma concentrations of C‐reactive protein in 2012/13.

**Result:**

Binary logistic regression models indicated that the odds of subsequent dementia increased by 1.19 for each additional health‐risk behaviour (95% confidence intervals: 1.04, 1.37, p = .014) after adjusting for age, sex, ethnicity, wealth, education, marital status, body mass index, coronary heart disease, hypertension, stroke, and depression. However, this association was not mediated by C‐reactive protein.

**Conclusion:**

Co‐occurring health‐risk behaviours were associated with higher dementia incidence up to 10 years later, underscoring the importance of modifying health‐risk behaviours for the prevention of dementia. Systemic inflammation did not explain the association between behaviours and dementia.

